# Valorization of *Salicornia patula* Duval-Jouve Young Shoots in Healthy and Sustainable Diets

**DOI:** 10.3390/nu16030358

**Published:** 2024-01-25

**Authors:** Irene Sánchez Gavilán, Daniela Velázquez Ybarzabal, Vicenta de la Fuente, Rosa M. Cámara, María Cortes Sánchez-Mata, Montaña Cámara

**Affiliations:** 1 Departamento Biología, Facultad de Ciencias, Universidad Autónoma de Madrid, Campus Cantoblanco, 28049 Madrid, Spain; irene.sanchezgavilan@estudiante.uam.es (I.S.G.); vdelafuente@uam.es (V.d.l.F.); 2 Departamento Nutrición y Ciencia de los Alimentos, Facultad de Farmacia, Universidad Complutense de Madrid, Plaza Ramón y Cajal, s/n, 28040 Madrid, Spain; daniela.velazquezyl@udlap.mx (D.V.Y.); rm.camara@ucm.es (R.M.C.); cortesm@ucm.es (M.C.S.-M.)

**Keywords:** halophytes, dietary fiber, organic acids, sustainable diets, healthy diets

## Abstract

The revalorization of natural resources in food production is increasing, and the effect of climate change is negatively affecting the production of conventional crops. In recent years, edible halophytes have received more attention due to their ability to tolerate a wide range of salinities. Thus, the use of halophytes that require less water and are strongly adapted to high-salinity soil and coastal areas can provide sustainable agriculture in certain areas. In addition, there is growing interest in the study of the possibilities that these species offer as foods due to their excellent nutritional profile and antioxidant properties. For that reason, the exploitation of plants adapted to these areas is nowadays even more important than in the past to guarantee food security in arid or semiarid salinized territories. The available data about the nutrients and bioactive compounds composition of many non-cultivated edible vegetables traditionally used in the Mediterranean area, such as Salicornia edible young shoots, are still scarce. With the aim of improving the knowledge on their nutritional value, the present study provides new data about the content of some compounds with biological activity, such as fiber and organic acids, in eight samples of young shoots of *S. patula* Duval-Jouve gathered in great mainland and coastal salt marshes in Southwest and Central Spain. Results showed that this vegetable can be considered a healthy food and a very good source of dietary fiber (4.81–6.30 g/100 g fw total fiber). Its organic acid profile showed oxalic, malic, citric and succinic acids. Oxalic acid was the major one, with mean values of 0.151–1.691 g/100 g fw. From the results obtained in this study, *S. patula* shoots could be recommended as an alternative source of fiber for healthy and sustainable diets in the general adult population with no risk of renal disease.

## 1. Introduction

A high dietary consumption of vegetables is globally accepted as a key point to reaching a healthy diet. Chronic diseases, such as obesity, cardiovascular disease, metabolic syndrome, and so on, are increasing in Western societies, and some modifiable factors may reduce the risk of these diseases, of which diet is one of the most important [1]. The increase in the presence of plant-based foods in the diet has been proposed to improve the health status of the population. In light of this fact, the recovery of autochthonous species in the diet is a valuable tool to achieve: (i) the diversification of plant-based foods in the diet; (ii) the intake of different nutrients and bioactive compounds from different species, which on the whole may act in a synergistic way; and (iii) the preservation of traditional dietary habits and biodiversity, which contribute to rural development.

These points are within the principles of the Mediterranean Diet, which encourages the use of a wide range of crops for cereals, fruits, and vegetables, not only cultivated products but also wild species, thus sustaining them together with local and traditional knowledge about their use. This dietary pattern has been widely postulated as a healthy model that may reduce the risk of several chronic diseases [2].

The WHO recommends a minimum daily intake of 400 g of vegetables and fruits as a population target. However, the consumption of vegetables and fruits by the Spanish population is below these recommendations, as has been shown in the different nutritional surveys and in the national health surveys [3]. One of the reasons to make this recommendation is the fact that fiber consumption in Western societies is insufficient, and its deficiency is directly linked to certain diseases. Fiber is a key group of compounds that should be present in the diet to provide important health benefits such as the regulation of intestinal transit, satiety, retardation of glucose and fat absorption, lowering cholesterol levels in the plasm and improvement of the gut microbiota. This involves positive effects against constipation, obesity, diabetes, dislipemia and cardiovascular diseases, as well as a reduction in the risk of some colorectal cancers [4,5]. To achieve these benefits, the recommendations for consumption of Total Dietary Fiber in adults, according to the European Food Safety Authority (EFSA), are 25 g/day, including a proportion of 3:1 for insoluble:soluble fiber, based on epidemiological studies that show protection against cardiovascular diseases. Providing the different mechanisms of action of the compounds included in the insoluble and soluble fiber fractions, the intake of both fractions is recommended [5]. The required daily fiber intake can be obtained from foods such as fruits and vegetables, whole grains, legumes, nuts, and others, or by eating foods enriched with fiber as a functional ingredient [6].

It Is well known that many leafy vegetables may have high levels of oxalic acid; this compound, when ingested in high amounts, is excreted in urine, causing hyperoxaluria, which leads to the deposition of calcium oxalate in kidney tissue or crystallization as calcium oxalate kidney calculus (nephrolithiasis). The minimal lethal dose of this organic acid for adults has been established as 5 g per day, which is a very difficult level to reach in a normal and balanced diet. However, people with a sensitivity to this kind of calculus should reduce the amount of oxalic acid in their diet to avoid this problem. Besides this, oxalate forms insoluble complexes with minerals such as Ca and, to a lesser extent, Mg in the gut; the insoluble complexes precipitate and are not absorbed, leading to a lower bioavailability of these minerals and also less absorption of oxalates. Thus, a high intake of these minerals is desirable to impair the negative effects of oxalic acid as both the urinary deposition of absorbed oxalic acid, and the reduction of mineral absorption [7].

The Food and Agriculture Organization of the United Nations (FAO) is concerned about promoting new models of agriculture using locally adapted management practices to provide sustainable agriculture and rural development. FAO highlights soil salinization affecting food security and remarks on “the importance of the selection of salt-tolerant crops and plants, including halophytes, which are able to grow well in such environments” [8].

Severe salinization of agricultural land due to the improper use of irrigation and fertilizers is reducing the area of arable land in the world [9]. Moreover, currently one-sixth of the world population inhabits arid or semiarid regions [10]; in these areas, soil salinization is a key issue with substantial impact on plant productivity, making fresh fruit and vegetable cultivation difficult and representing a serious threat to food security [11]. Harvesting salt-tolerant crops can provide an economic resource in soils with severe salinization. To survive in these saline environments, plants have developed different adaptive mechanisms, including altered growth patterns, osmotic adjustment, and ion homeostasis [12].

Halophytes represent about 1% of the world’s flora and are found in salt deserts and saline areas such as beaches, salt marshes, and mangroves. The majority of the crop and forage species used in modern agriculture are salt-sensitive (glycophytes) and can handle only a very limited concentration of salt in their growth media. In fact, halophytes can tolerate a wide range of salinities, even beyond seawater concentration (approx. 500 mM NaCl), and can withstand harsh conditions, such as drought and intense UV radiation [13]. For these reasons, there is a growing economic interest in the study and production of halophytic plants for food uses [14,15,16], as wild halophyte plants have been traditionally used since ancient times in different areas worldwide, where people living in salinized areas have adapted to the surrounding vegetation.

A health concern regarding the use of halphytes as foods is related to their high Na content. While most vegetables contain a very low Na amount (about 25 mg/100 g or less), many halophytes grow in salinized soils and may accumulate Na up to 1 g/100 g in young shoots and other edible parts [17]. This may be inconvenient, as dietary Na levels should be low to avoid hypertension and reduce cardiovascular risk. For this reason, a maximum intake of sodium of 2 g/day has been recommended by EFSA (2017) [4]. Despite this fact, the use of halophytes to replace common salt in foods such as salads or others may be a good strategy, just as fiber and other nutrients are improved in the diet.

Among halophyte plants, the Chenopodiaceae family is the most, with *Salicornia* species being the most frequently eaten [18,19]. These species grow and have been used as traditional food and in folk medicine in many different regions, particularly in Mediterranean countries such as Spain, Portugal, Italy, and Tunisia [10,11]. In England, France, the USA, and Australia, they were also known as “herbe de Saint Pierre”, anglicized in the mid-16th century as samphire, and consumed in situations of extreme need such as famines [20].

In the particular case of *S. patula*, it is well known as an authoctonous species from the Iberin flora, with an essential presence in the habitat of Community Interest No. 1510, “Mediterranean salt steppes”, which is mainly present in the autonomous communities of Andalusia, Castilla la Mancha, and Murcia (Spain) in the Natura 2000 European network [21,22]. As with other species of Salicornia, it has been traditionally used as a food in many different regions of the Mediterranean area where it grows. As an example, *S. europaea* L. is often consumed as a salad dressing or as a side dish for fish [19], and it is currently listed as a traditional vegetable in the Apulia region (Italy), where also cultivation attempts of *S. patula* have been made along Lesina lagoon [23,24]. Recently, in Setubal (Portugal), a different halophyte, *S. perennis*, was incorporated as a food ingredient into crackers, like savory snacks, to replace sodium [25]; it has also been powdered, dried, and incorporated into bread to reduce the same amount of sodium [26]. Furthermore, the use of dried *S. ramosissima* as a salt substitute shows great potential as an alternative replacement for sodium-based additives, and their sprouts are steamed and subsequently added to salads or other stews in the Valencian region (Spain) [27], and *S. patula* is also described for food use in Eastern Spain [20].

Thus, looking to safeguard economic and environmental sustainability, the remarkable tolerance of *S. patula* to salinity, drought and high temperatures and its low input needs for cultivation make this plant a resilient crop for the immediate future [28].

In relation to health benefits, some studies have confirmed that *S. patula* is a good source of macronutrients, essential minerals, and some bioactive compounds such as polyphenols, fatty acids, and vitamins [24,29,30,31]. However, there is a lack of knowledge about the fiber content and some bioactive compounds in the edible parts of this species.

With the aim of improving current diets in a healthy and sustainable way and the valorization of *S. patula* bioactive compounds, the present study is focused on the content of fiber and organic acids in the young shoots of wild *S. patula* samples from different geographical locations in Spain. This knowledge will contribute to revalorizing these wild halophyte species and promoting their crop adaptation for culinary uses, especially in semi-arid regions where the drought seriously threatens the agriculture productivity and compromises food security.

## 2. Materials and Methods

### 2.1. Plant Material

Young shoots of wild *S. patula* were collected in their saline natural habitats within the Iberian distribution ranges focused in southwest and central Spain. Eight different geographical locations in Spain, five of them; S1 to S5 are located close to Tinto River area (southwest Spain) and the other three S6 to S8 are located at the center of the Iberian Peninsula, as is shown in Table 1.

Plant materials were collected at their optimal status for food consumption: succulent and tender, young shoots. Samples were processed as follows: whole plant was carried to the lab on fridge at 4 °C. Once in the lab, samples were cleaned and young edible shoots were dried in oven at 95 °C and homogenized (dry powder plant material). Then, samples were stored in airtight jars at room temperature and darkness. All analytical determinations were made in triplicate for each sample coming from each collection area.

### 2.2. Moisture Determination

Moisture was determined by desiccation in an oven at 105 °C for 24 h until a constant weight was reached [32].

### 2.3. Fiber Determination

The insoluble (IF), soluble (SF), and total dietary fiber (TDF) contents were determined on dried plant material by the enzymatic gravimetric method AOAC 993.19 (FS) and 991.42 (FI) [32,33]. To obtain the insoluble fiber, samples were subject to enzymatic digestion with α-amylase, protease, and amyloglucosidase (Sigma-Aldrich, St. Louis, MO, USA) in order to hydrolyze the protein and starch present in the samples. The liquid obtained was filtrated on Pyrex crucibles with a number 2 filter plate. Insoluble fiber residue was dried in an oven at 100 °C and then weighed. The filtrate was stored in a 500 mL flask with the addition of 400 mL of 96% *v*/*v* ethanol and precipitated from one day to the next. Then, it was filtered again through crucibles with the same conditions to obtain the soluble fiber residue. In both residues, the content of protein and ash was determined, and the content corresponding to insoluble and soluble fiber was calculated (with the subtraction of protein and ash).

### 2.4. Determination of Organic Acids

Individual organic acids were determined by HPLC, based on procedures optimized by [33] and applied to different types of plant samples by [34,35,36]. Extraction was performed with 0.5 g of dried sample in 25 mL of 4.5% m-phosphoric acid and analyzed using an HPLC-UV methodology.

**Figure 1:**
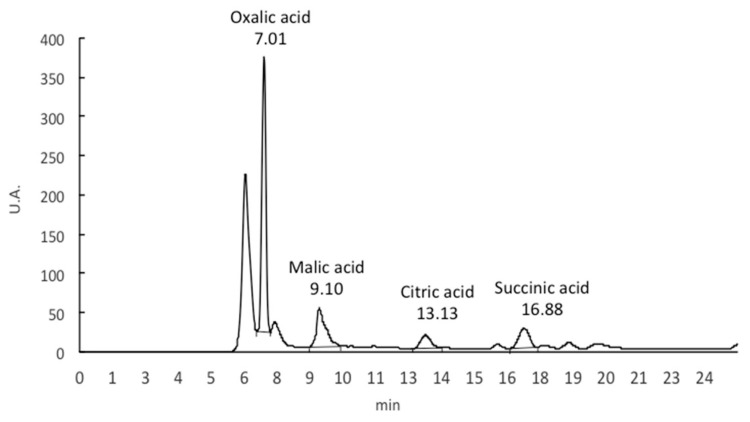
Chromatogram obtained for organic acids in *S. patula* Duval-Jouve edible shoot samples.

The HPLC equipment was a liquid chromatographer equipped with an isocratic pump (model PU-II, Micron Analítica, Madrid, Spain), an AS-1555 automatic injector (Jasco, Tokyo, Japan), a Sphereclone ODS (2) 250 × 4.60, 5 mm Phenomenex column (Torrance, CA, USA), a UV detector (Thermo Separation Specta Series UV100, San Jose, CA, USA) working at 215 nm. The mobile phase was 1.8 mmol/L H_2_SO_4_ (pH 2.6) at 0.4 mL/min flow rate. Data were analyzed using Chromonec XP software (Micronec, Madrid, Spain). Identification was performed comparing retention times with those obtained from commercial pure standards of oxalic, malic, citric, and succinic acids. Quantification was based on the UV signal response, and the resultant peak areas in the chromatograms were plotted against concentrations obtained from standards (Figure 1). Organic acids contents in *S. patula* samples were expressed as mg/100 g of fresh plant material.

### 2.5. Statistical Analysis

Statistical analysis was performed using Statgraphics 18.0 software. Mean and standard deviations were calculated. The data were transformed logarithmically after verifying normality with the Shapiro–Wilk test (*p* > 0.05). To test the possible differences between three or more groups, they were compared using an ANOVA analysis of variance. Bonferroni corrections between means were calculated only if an F test was significant at *p* < 0.05.

## 3. Results and Discussion

Table 2 shows the values obtained for the moisture, fiber, and organic acid content (fresh weight, fw) of the fresh *S. patula* shoots belonging to the eight populations analyzed in this study.

*S. patula* edible shoots had a moisture content with few differences among the analyzed samples between 89.8 and 91.3 g/100 g, which confers them tender and succulent characteristics. The *Salicornia* sample collected in Valladolid, in the center of the Iberian Peninsula, stood out with the highest moisture value (91.3 g/100 g), whereas sample 2 from “Moguer”, Huelva, presented the lowest moisture content (89.8 g/100 g) [29,30].

**Figure 2:**
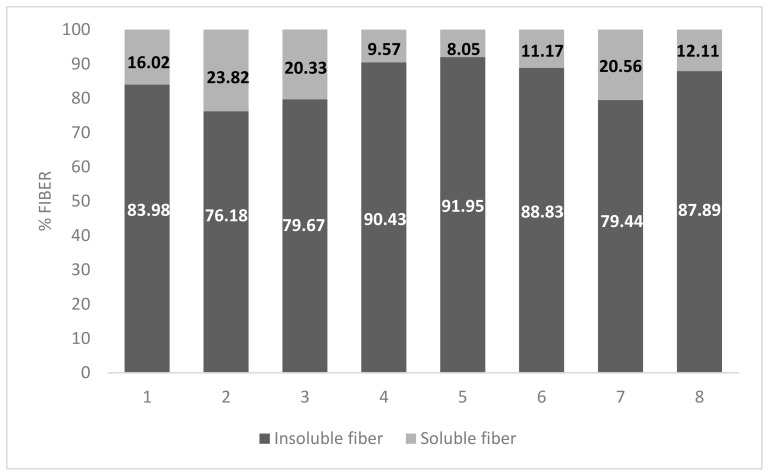
Proportions of insoluble and soluble fiber in *S. patula* Duval-Jouve fresh samples (g/100 g dw).

Regarding fiber content, the *S. patula* fresh shoots studied contained between 4.8 g/100 g fw and 6.6 g/100 g fw (53–73 g/100 g dw), with insoluble fiber being the predominant fraction, with an average proportion of 84.79% (Figure 2). Few significant variations can be found in the values reported, with the exception of soluble fiber, where S2, S3, and S7 stood out with higher values than other samples. These variations cannot be attributed exclusively to a single factor, but they may be a product of the natural variability found in biological samples.

This is the first report on the fiber content of *S. patula* young shoots. Despite this variability, the comparison with other edible halophyte species shows higher values for *Suaeda fruticosa* Forrsk shoots with 10 and 12 g/100 g fw, similar to *Arthrocnemum macrostachyum* Moric. and *Halocnemum strobilaceum* Pall (*M*)*. Bieb*., from Tunisia, with 10.1 and 9.1 g/100 g [37].

On the other hand, other halophytes native to Portugal, such as *S. perennis alpini* (Lag) *Castroviejo*, have been reported to contain 15.3% of total dietary fiber, and *S. ramosissima* J. Woods presented values of 11.2% dw, both of which are being considered as fiber-rich vegetables [11]. *S. patula* young shoots analyzed in this study presented higher values of fiber than both species.

Thus, from the point of view of fiber content, *S. patula* edible parts may be a very good contributor to the diet. The contribution of 4.8–6.6 g of fiber per a portion of 100 g of *S. patula* young shoots is higher than that of other leafy vegetables and could cover up to 19–25% of the recommended intake of total dietary fiber per day, which is a relevant amount. When comparing fiber levels with other vegetables [38], it can be seen that most leafy vegetables present 2–4 g/100 g fiber; *S. patula* provides a higher amount of fiber than most conventional fresh vegetables, being only surpassed by artichokes (about 9 g/100 g). Therefore, the recovery of this vegetable as a food may be a key tool to improve fiber intake in the diet and thus the health status of the population. Regarding the proportions of soluble/insoluble fiber, the analyzed samples present high proportions of insoluble fiber contributing to the effects on intestinal transit and satiety, among others. For those reasons, and according to Regulation (EC) No. 1924/2006 of the European Parliament and of the Council of 20 December 2006 on nutrition and health claims made on food, *S. patula* young shoots could be claimed as “sources of fiber” for providing more than 3 g fiber/100 g fresh plant, and in many cases, as “high amounts of fiber” for providing more than 6 g fiber/100 g [39].

Table 3 shows the organic acid profile and content of the analyzed samples. The identified organic acids were oxalic, malic, citric, and succinic acids. As expected, oxalic acid is predominant, as occurs in other leafy vegetables, with wide variability between 1.73 mg/100 g dw and 18.34 mg/100 g dw, corresponding to 0.151 g/100 g fw and 1.69 g/100 g fw.

Although it has quite low toxicity (with 5 g as the minimal lethal dose for an adult), this compound may reduce dietary calcium bioaccessibility through the formation of an insoluble complex as well as the formation of calcium oxalate kidney calculus [40]. For those reasons, some authors recommended a molar oxalic acid/Ca ratio not higher than 2.5 in the foods to avoid this effect [41,42].

To calculate the oxalic acid/Ca ratio, the data on the calcium content previously obtained in the analysis of the same Salicornia samples by Inductively Coupled Plasma Mass Spectrometry [29] were used. The ratios obtained are presented in Table 3, showing a ratio higher than 2.5, higher than nutritionally desirable. Samples collected from the “Tinto River”, in Southwest Spain (S1 to S5) showed a greater variety and amount of organic acids, with oxalic acid standing out, and the oxalic acid/Ca ratio is higher in these samples than in the material from Central Spain (S6 to S8). The differences in oxalic acid/Ca ratio between young shoots of *S. patula* could be related to the different elemental compositions of the soil. Calcium content is very low in the Tinto River area and higher in soils from central Spain [29].

It can be seen that, although this species should be considered an oxalic-rich vegetable, even samples with the highest amounts of oxalic acid (S3) would not provide more than 1.6 g of this undesirable compound in a standard 100 g portion. Very high amounts of this plant (about 250 g) that are hardly eaten by humans would be necessary to reach toxic doses. Thus, for the general population and with a reasonable consumption (portions lower than 100 g) within the context of a diversified diet, the oxalic acid content of this vegetable would not represent a potential risk for human consumption, according to the results presented. However, given that the content of this compound is high and the low oxalic acid/calcium ratio, young children and people who easily form kidney oxalate calculus should avoid the ingestion of oxalic acid-rich species. Another alternative could be boiling to reduce oxalic acid content by dissolution, as it is done with other vegetables [7,31]. However, highly vulnerable people should avoid this kind of vegetable.

Other minor organic acids present in *S. patula* were malic, citric, and succinic acids but they did not appear in all samples analyzed. Malic acid is present in five of eight analyzed samples, with ranges between 0.157 and 0.979 g/100 g dw. Other authors suggested malic acid accumulation in other *Salicornioideae*, like *S. perennis* from “Mira” (Portugal), under stressful situations, increasing in vegetative stages with high salinity and lower water content [43].

Citric acid was detected only in four of eight samples, with values between 0.201 and 0.719 g/100 g dw. Our results were significantly lower compared to other studies [44], reporting higher content: 8.60 mg/100 g in *Salicornia ramosissima* form “Ría Formosa” (Portugal) and 6.98 mg/100 g dw in *Sarcocornia perennis* (Miller) A. J. Scott, and were similar compared with those of *Sarcocornia fructicosa* (L) A. J. Scott and *Salicornia bigelovii* Torr. [15,45]. In some Australian halophytes, *Atriplex nummularia* Lindl, *Suaeda arbusculoides* L.S.Sm., and *Sesuvium portulacastrum* (L) L., higher citric acid values, between 3.33 and 23.9 g/kg dw, have been reported [46].

Succinic acid is present in four of eight, with a content between 0.109 and 0.785 g/100 g dw. Succinic acid in other halophytes like *S. perennis* shows higher content in the vegetative segment than the fruit segment in three different locations of “Ría de Aveiro”, with an average of 0.17 and 0.51 μg/mg. Other authors suggested that succinic acts like an osmoprotectant in response to high-salinity environments [47].

Organic acids, either total content or individual profile, can have a significant effect on the sensory, aroma, and flavor of plant-derived foods and, subsequently, consumer acceptance [48]. All these components contribute to reinforcing the interest in this vegetable in the diet. The high presence of sodium, in levels of 1–1.7 g Na/100 g [29], makes it a candidate to be used as a substitute for salt in salads or other different food products, where they are a source of salted flavor at the same time that they provide fiber and other nutrients.

It should be pointed out the importance of a widely diversified intake of many vegetables in the diet, in which all different, either cultivated or wild, autochthonous edible species may be included. In the case of vegetables rich in oxalic acid and/or sodium species such as *S. patula*, boiling is preferable for vulnerable populations (e.g., young children, people who need to control blood pressure), while people at risk of renal disease or infants should avoid these vegetables.

## 4. Conclusions

In conclusion, the studied young shoots of wild Spanish halophyte, namely *S. patula*, can be considered a healthy food and a very good source of dietary fiber, with the insoluble dietary fiber fraction being the most important one, contributing to improving the nutritional quality of the current diets, even as a common salt substitute providing additional nutrients. The main organic acids present are oxalic, malic, citric, and succinic acids. Oxalic acid was the major one; the contents found would not represent a health problem in an occasional intake of an edible shoot of *S. patula*; however, young children and people who easily form kidney oxalate calculus should avoid the ingestion of oxalic acid-rich species. Boiling could also be advisable to reduce sodium and oxalic acid levels. Thus, *S. patula* could be considered a good alternative to other conventional vegetables for the diversification of sustainable diets with high nutritional potential.

## Figures and Tables

**Table 1:** Description of sites in Spain where *S. patula* Duval-Jouve young shoots were collected.

Sample ID	Site	Grid Reference MGRS
S1	Huelva, La Rábida	29SPB8320
S2	Huelva, Moguer	29SPB7729
S3	Huelva, El Terrón	29SPB4717
S4	Huelva, San Juan del Puerto	29SPB9230
S5	Huelva, Monumento a Colón	29SPB8220
S6	Valladolid Aldeamayor de San Martín	30TUL6196
S7	Madrid, Colmenar de Oreja	31TVK5143
S8	Toledo, Laguna larga de Villacañas	30SYH0928

**Table 2:** Total, insoluble, and soluble fiber contents (g/100 g fresh weight) in *S. patula* Duval-Jouve samples, and contribution of each fraction to total dietary fiber content (results presented as mean ± SD).

Sample	Moisture	Insoluble Fiber	Soluble Fiber	Total Fiber
S1	90.121 ± 0.621 ^a^	4.417 ± 0.101 ^a^	0.841± 0.323 ^a^	5.269 ± 0.336 ^a^
S2	89.876 ± 1.123 ^b^	4.800 ± 0.441 ^ab^	1.409 ± 0.134 ^b^	6.301 ± 0.438 ^b^
S3	90.725 ± 0.731 ^a^	4.603 ± 0.614 ^ab^	1.114 ± 0.190 ^b^	5.782 ± 0.811 ^a^
S4	91.041 ± 0.775 ^ab^	4.612 ± 0.140 ^a^	0.488 ± 0.182 ^a^	5.104 ± 0.276 ^ab^
S5	90.183 ± 1.411 ^a^	5.660 ± 0.312 ^b^	0.519 ± 0.081 ^a^	6.185 ± 0.290 ^a^
S6	91.290 ± 1.012 ^ab^	5.682 ± 0.129 ^a^	0.793 ± 0.073 ^a^	6.307 ± 0.273 ^ab^
S7	90.763 ^a^ ± 1.356 ^a^	3.996 ± 0.181 ^a^	1.034 ± 0.372 ^b^	5.038 ± 0.543 ^a^
S8	91.111 ± 0.813 ^ab^	4.229 ± 0.163 ^a^	0.568 ± 0.051 ^a^	4.812 ± 0.221 ^ab^

In each column, different superscript letters mean statistically significant differences (*p* < 0.05) compared by Shapiro–Wilk test.

**Table 3:** Organic acids contents (g/100 g fresh weight) in *S. patula* Duval-Jouve samples (results presented as mean ± SD).

Sample	Oxalic Acid	Malic Acid	Citric Acid	Succinic Acid	Oxalic Acid/Ca
S1	0.8224 ± 0.061 ^a^	0.016 ± 0.002 ^a^	0.072 ± 0.008 ^b^	0.033 ± 0.008 ^a^	8.132 ± 0.241 ^a^
S2	1.060 ± 0.018 ^b^	0.015 ± 0.001 ^a^	0.020 ± 0.003 ^a^	0.012 ± 0.004 ^b^	11.381 ± 1.313 ^b^
S3	1.691 ± 0.007 ^b^	Nd	Nd	Nd	34.006 ± 3.711 ^ab^
S4	1.187 ± 0.067 ^b^	0.087 ± 0.053 ^b^	Nd	0.009 ± 0.003 ^a^	11.082 ± 1.192 ^a^
S5	1.533± 0.045 ^b^	0.016 ± 0.011 ^a^	Nd	0.077 ± 0.001 ^b^	7.581 ^a^ ± 0.237 ^a^
S6	0.151 ± 0.023 ^ab^	0.024 ± 0.071 ^a^	Nd	Nd	6.689 ± 1.052 ^a^
S7	0.758 ± 0.013 ^a^	0.019 ± 0.017 ^a^	0.004 ± 0.000 ^a^	Nd	8.061 ± 1.133 ^a^
S8	0.877 ± 0.555 ^a^	Nd	0.003 ± 0.000 ^a^	Nd	4.763 ± 0.920 ^ab^

In each row, different superscript letters mean statistically significant differences (*p* < 0.05) compared by Shapiro–Wilk test. Nd = non detected.

## Data Availability

Data is available only upon request. Tables included in the manuscript show all the data.
